# REPORT ON SYSTEMATIC EVIDENCE REVIEW

**DOI:** 10.1093/geroni/igz038.2745

**Published:** 2019-11-08

**Authors:** Howard Fink

**Affiliations:** Minneapolis VA Health Care System, Minneapolis, Minnesota, United States

## Abstract

During the workshop, results from a systematic evidence review prepared by an Agency for Healthcare Research and Quality Evidence-based Practice Center to inform the workshop were presented, focusing on long term osteoporosis drug treatment. This talk will describe the goals of the systematic review, which addressed four questions: (1) What are the effects of long-term (>3 years) osteoporosis drug treatment versus control on risks of incident fractures and harms; (2) Do effects of long-term osteoporosis drug treatment vary as a function of patient, bone, or osteoporosis drug characteristics; (3) Among individuals receiving osteoporosis drug treatment to prevent fracture, what are the benefits and harms of continuing versus discontinuing treatment (e.g., osteoporosis drug holiday); and (4) Do outcomes of drug discontinuation or drug holidays vary as a function of patient, bone, or osteoporosis drug characteristics?

